# Akesius; a Glance into the Ethical Relations of Medicine

**Published:** 1845-01

**Authors:** 


### Art. XII.

*Akesios. Blicke in die ethischen Beziehungen der Medicin.* Von K. F. H. Marx.—*Göttingen*, 1844.

*Akesius; a Glance into the Ethical Relations of Medicine.* By K. F. H. Marx.—*Göttingen*, 1844. 12mo, pp. 152.

Akesius, the healer, according to Sprengel, was one of the names given to the demigod, known to the Egyptians as Harpocrates. His birth at the winter solstice was symbolical of the powerlessness of the sun at mid-winter; but indicated also the hope of more genial warmth with its return along the ecliptic. To the sick he was at once the emblem of their debility, and of their hope of restoration to health. He was represented also with his finger on his lips, to symbolize that sacred secresy which the initiated were bound to maintain respecting the mysteries of their calling.

Professor Marx, in terming his little volume Akesios, refers only to the first-mentioned symbol of the god, for he does not treat of the means or methods of curing disease. He has chosen for his theme (to use his own words) that which springs from the heart and not from the head; it is the morals, the ethics, the spiritualism of medical theory and practice of which he professes to discourse.

The fundamental principles of medical ethics can be none other than the fundamental principles of Christianity, namely, an unqualified love and reverence of God, and an unselfish love and honour of humanity. With these principles, guiding alike his scientific inquiries and his daily duties, the medical practitioner has scope and opportunities for happiness presented to him equalled by those of few other pursuits, and surpassed by none. We doubt much if even the clerical profession offers a wider field for doing good, or more numerous opportunities for high communion with God. The object of the physician's professional studies is man; and man he studies through all nature. Thus, indeed, it has happened that medicine has been the prolific root of all the varied branches of natural philosophy, and that physicians have ever been the most successful and celebrated cultivators of the accessory sciences. We could name men now living and distinguished in almost every branch of human knowledge whose primary education was medical.

The tendency, in fact, of this wide range of mental labour has necessarily been to elevate and give a uniform development to the faculties of the physician. It has raised him to an Alpine height of intellectual vision, from whence he can look down on the details of this gigantic machine of the universe, and see in incessant activity that wonderful system of poises and counterpoises by which the MASTER-MIND regulates his own creations. From the same intellectual eminence he can also survey the battle-field of the excellencies and infirmities of poor humanity. How easily the observant eye of the physician penetrates the veil of modesty or vanity man throws over his acts and motives during sickness! He witnesses within one short day heroic self-denial, surpassing fortitude, utter abasement, the most servile fear, and pride grasping its ragged mantle of appearances, and vainly attempting to hide its ulcers and deformities, even to that last solemn moment when time joins eternity.

The moral results of medical studies are not less than the merely intellectual. They appear, however, in the physician rather as the smooth, placid, ever-flowing stream of active benevolence, than as a high-wrought enthusiasm or daring undertakings. A thorough knowledge of his own imperfections and infirmities checks the one and forbids the other; while a deep conviction of the power of truth continually cheers him to seek and use her as the means of honouring God and perfecting humanity.

Embued with such sentiments, the physician cannot but be impressed with the dignity of his pursuits; he cannot conceal from himself that his mission is to ameliorate the "primal curse;" that he is the special messenger of Providence to suffering man; prolonging his days, relieving his sufferings, ministering to his happiness. Some practitioners may see and appreciate all this less distinctly than others, but there are few who will not feel in some degree that we are expressing sentiments in harmony with their own.

General and medical literature have in all ages presented proof how these sentiments have impressed thinking minds. Homer terms Machaon an *ἰσοθεος φῶς*, a divine light. Cicero expressed the same sentiment more distinctly: "In nulla re proprius homines accedunt ad deos, quam salutem hominibus dando." It is impossible to peruse the ethical portions of the Hippocratic writings without feeling their moral grandeur. In the book 'De Medico,' it is asserted that the truly philosophic physician is godlike; using the identical term (*ἰσοθεος*,) applied by Homer to Machaon, and adding, "that indeed he differs little from the gods." The list of moral qualities given as requisite to the right formation of the medical character, exhibits a purity of moral feeling and sentiment unsurpassed by any modern author. The notions respecting the dignity of the medical art were carried even to a ridiculous excess, for Menecrates of Syracuse maintained that practitioners of it ought actually to be worshipped as gods. Philip of Macedon, to punish this presumption, took Menecrates at his word; and, instead of feeding him with earthly food, offered him incense. Further corroboration, if that were necessary, of the elevated sentiments respecting medicine and its professors current in ancient Greece, is given by the correspondence of Hippocrates; and this whether that correspondence be genuine or not. Artaxerxes is represented as offering to Hippocrates (through Pœtus, prefect of the Hellespont,) as much gold as he may desire, and the highest honours of the Persian nobility, in return for his services as physician-general to the Persian army, then devastated by a pestilential disease. Hippocrates, in reply, contemns the proferred riches and honours, and in spirited terms refuses to assist the enemies of his country. To his friend Demetrius he writes, "The king of the Persians called me to him, not knowing that moral precepts had greater weight with me than gold." And when he was sent for by the citizens of Abdera to the relief of Democritus, in his reply to their letter, he repeats his principles of ethics: "Riches," he says, "do not consist in stores of gold, but in the practice of virtue." In the same letter he compliments the Abderites for taking so deep an interest in the health of Democritus; observing, "certainly, happy is the people who esteem good men to be their best bulwarks, and prudent counsel their strongest walls and towers." With all this wisdom, Hippocrates was a man. He grumbled, as modern doctors grumble, at the ingratitude and unreasonableness of his patients. He roundly asserted in his old age, that he had during life got far more blame than praise—forgetting the almost divine honours that had been paid him, and that humanity was an infirm thing, full of ignorance and prejudice. But in every age wise old men have forgot the true philosophy, of taking men as they find them. Accustomed to homage, they demand obedience rather than persuade to it. Their expectations are really inconsistent with their experience, and they are therefore disappointed.

There are many examples in modern literature like to those we have noticed—men made up of mighty intellect and human frailty, but not the less to be honoured and esteemed, for they were good men. Sir Thomas Brown, the author of the 'Religio Medici,' was one of these. A modern essayist, writing of him observes (pointedly with reference to our present remarks): "What the philosopher of Norwich considered miraculous, was probably this, that he had escaped from 'Pyrrho's maze,' and had never been contaminated in 'Epicurus' stye;' that he had neither striven for place among the 'wrangling crew,' nor sought to make his way with the sordid herd; that he had not sold himself to the service of Mammon; but in mature years, and with deliberate judgment, had chosen a calling in which he might continually increase his knowledge and enlarge his views, and entertain a reasonable hope that while he endeavoured to relieve the sufferings of his fellow-creatures and discipline his own mind, the labours wherein his life was past would neither be useless to others nor to himself."

Professor Marx introduces us to several such departed spirits as the author of the 'Religio Medici,' in a manner singularly quaint and attractive; for he discusses the ethical topics of medicine in a series of letters to Steiglitz, Cheyne, James Gregory, Hallé, Lettsom, Pinel, Mead, Boerhaave, &c., holding correspondence with them in the grave, as if they had not drunk of Lethe, and mortal affairs still had an interest with them.

> "Circumstant animæ dextrâ lævâque frequentes.
> Nec vidisse semel satis est: juvat usque morari,
> Et conferre gradum."

The first letter is to a friend the author has but recently lost:

"To Steiglitz. Since the 31st of October, 1840, when you said farewell to the earthly, my days, and not my writing-paper, have had a margin of mourning. At the unexpected news of your departure hence, I felt that the death of a friend rends the heart, that the descent of a great man to the grave is a disruption of nature. Three years have sped on since then, amid a variety of feelings, but my soul is still agitated as if I had lost you but yesterday, while I see more clearly the greatness of my loss. To distinctly recall the many years I lived with you in a close interchange of thoughts and feelings, and to celebrate a resurrection-festival of your love, I had begun your memoirs, as a monument to your memory; but, although so noble the material, the chisel that should have worked out the precious marble was useless in my trembling hand. But yet truly the world requires not my delineation, since to contemporaries you were a model of excellence, and to posterity your own works remain. To myself, however, you appear as a dear father or teacher, extending your hands to bless me. Why is it that to-day I invoke your name more loudly than usual? Because I intend to discourse of physicians who consecrated alike their soul and strength to pure humanity. Your modesty will not be offended if, grateful for that, I first discourse of you."

The great merit of Steiglitz consisted, according to his mourning friend, in the rare union of genius and experience. He was bold, too, for truth, and ever *called things by their right names*. How great are the men who thus name things! and necessarily great, because they set the right objects of toil before them, and see the shortest way to their attainment. These last-mentioned merits belonged also Steiglitz, and with them, freedom from envy, and a tolerant spirit.

The next letter is to Peter de Apono. "Famous Peter, permit me to thou thee, for thou wrotest in Latin and art used to be thoued," is the exordium of this epistle. Petrus de Apono was condemned after his death in 1316 by the inquisition, and his poor remains were ordered to be burnt; but a faithful female friend took his body from its grave and hid it; so that vengeance could only be wreaked on his effigy. People supposed that he was a sorcerer, and had learnt the seven liberal sciences by means of seven familiar spirits that he kept close prisoners in a crystal vase. He could also, happy man! make the money he spent come again into his pocket. Professor Marx congratulates his "dear Peter" on being more fortunate as to the mode of his departure from earth than Daniel of Gadden, who having collected various specimens for a museum of natural history (stuffed snakes and the like), was seized by the mob as a sorcerer, tortured, torn to pieces, and his intestines strewed about the street. This happened at Moscow in 1682. The ignorant ferocity and ingratitude of the populace towards physicians has been seen in our own day; the *Colonna infame* at Milan would not have been a solitary memorial of a paroxysm of public insanity if in 1832 the government of Russia had been less despotic.

The epistle to Dr. George Cheyne exhibits him as a teetotaller and a man of peace. The following quotation from his "account of himself," proves that he was as good a hydriatic as Priessnitz himself.

"I firmly believe and am as much convinced as I am of any natural effect, that water-drinking only will preserve all the opulent healthy from every mortal distemper; and that a diet of milk and seeds, with water-drinking only, duly continued and prudently managed, with proper evacuations, air, and exercise, is the most infallible antidote for all the obstinate diseases of the body and distemperature of the mind," &c.

Professor Marx assures Dr. G. Cheyne that when he treats on dietetics his principles shall be few and simple. Health he will maintain to be a virtue; serenity of mind a duty; that disgust at being sick cannot be too deeply impressed on the mind. The "*valere aude*," he would propose for adoption as a general salutation.

The epistle to Hallé sets forth the patience and benevolence of the conscientious practitioner. Hallé visited Malesherbes in prison and drew up the petition for the life of Lavoisier. If the stones of Paris could speak they would say, "Hallé wet us with his tears." Professor Marx describes vividly the painful and harassing nature of the duties of the physician in seeing suffering he cannot alleviate; ties broken he cannot keep unbroken; domestic griefs he cannot console. And while all look to the physician for solace none share his woes: "For the dying," Professor Marx observes, "there is an euthanasia; for the sorrowing, visits of condolence; but who cumbers himself about the mourning mediciner?" Many, we boldly answer, if, feeling himself the ministering messenger of Providence to afflicted man, he have done his duty cordially, earnestly, and well as his poor powers have permitted. There is no sweeter reward to the sick and sorrowing practitioner than the heartfelt condolence of grateful and affectionate patients, especially if they be poor. Tears are shed for his illness; sympathy expressed for his losses; and the prayers of the needy for him assuredly reach the throne of God. "Blessed is he who considereth the poor; the Lord shall strengthen him upon the bed of languishing."

The next letter is addressed to Dr. James Gregory, and discusses principally the literary education and attainments of the physician. The names of several medical poets are enumerated, those of Akenside and Darwin being, however, omitted. The titles of several proposed odes, catechisms, ballads, &c. are given with some satirical effect, as for example: "The Superfluousness of Technical Expressions, a Sermon; The Mutual fond Affections of Physicians, a Dream; The Friendships of Physicians, a Myth; The unexpected Meeting of two Physicians at the Patient's Bedside, a Physiological Interlude; On the Efforts made to become a domestic Physician, an Heroic Poem; On the Embarrassments of having neither Patients nor Friends, a Ballad;" and others similar.

A letter to Albert Thaer is followed by an amusing collection of aphorisms and aphoristic puns. Several of the latter, for obvious reasons, lose all their point in translation. We shall conclude our notice of this charming little volume with a small selection from these aphoristic sentences. We number them, for the sake of easy distinction:

1. "Time is the most experienced doctor; patience is the gentlest medicine.

2. "Thanks for a merciful punishment come as sincerely from the heart as a risus Sardonicus.

3. "Physicians are bred to be honorary members of all humane societies.

4. "Every vein, except a silver vein, returns to the heart.

5. "The physician like the diplomatist, must tread softly.

6. "He who makes the cerebellum to be the seat of instinct cannot say truly that his arbor vitæ (tree of life) is the tree of knowledge.

7. "The conscientious physician is just as guilty of the death of his patient as the still-born infant of the death of its mother.

8. "How many have nothing more sacred within them than the os sacrum!

9. "The prescription of a pulvis (dust) is not unfrequently a memento mori.

10. "It is with many friendships as with the teeth, when the enamel is gone they give pain.

11. "Lunar caustic is white but it darkens the skin; so the hypocrite laughs openly, but in secret blackens the character.

12. "To gild the pill is out of fashion, but not to gild the palm.

13. "Tournaments have ceased; but the physician ever jousts with antagonists having visors down, and a death's head on their shields.

14. "Man may learn the prudence of yielding sometimes, from this, that a stiff-neck is considered a disease.

15. "Physicians risk their lives often, most men, even soldiers, seldom.

16. "An autobiography unrolls a mummy before a glass.

17. "He who has no theory, cannot be practical.

18. "The plunder of a church is severely punished, that of the intellect goes free.

19. "Many stick to their calling as they would to a tread-mill; they often try to mount, but never rise.

20. "The rich have an idiosyncrasy, not against devils, but against poor devils.

21. "A man will remember you longer for giving him gold than for giving him health.

22. "Men must treat their acquirements as Tippoo Saib his pearls; ever seek after better.

23. "Pleasant words, like honey in an electuary, unite dissimilar things together.

24. "The physician has more need of a chart than of a map; for it is much more important to him to know what to avoid, than where to stop.

25. "The worst inheritance is an hereditary disease.

26. "Great men are the lighthouses of humanity.

27. "Emptiness is contemptible; even children will not play with deaf nuts.

28. "Metastatic diseases, as for example, gout and rheumatism, must be treated like vagabonds, with bread and water.

29. "The hop-dances, are to the lungs, death-dances.

30. "England for ever! who knows not himself knows nevertheless the English plaster and the English salt.

31. "To the unfortunate the heart is as a gaping wound, from which the blood flows unstaunched.

32. "The best medical police consists in dignified humanity. Through this the relations of medicine to science, to the state, to the public, to the colleges are most readily developed. High morals, energetic habits, and a well-grounded education are the surest means of success." (pp. 85, &c.)

With this wisdom we close our extracts; not without a hope that the doctrine of the last aphorism may, in England at least, be applied to practice, and the profession generally be to society what its individual members have been to individuals, an *ἰσοθεος φῶς*, a divine light, irradiating, invigorating, and perfecting our social condition.

We have before recommended the works of Professor Marx to the student of German language and literature. The terseness and elegance of his diction are equalled only by the charm of his sentiments. Besides he is well acquainted with our language, our literature and our institutions. His ethics, we need not say, are in every way British, refined and illustrated by the operation of his own mind.

---

